# Supplementary material for: Impact of supply chain disruptions and drug shortages on drug utilization: A scoping review protocol
Source: PLoS One. 2024 Nov 1;19(11):e0313298. doi: 10.1371/journal.pone.0313298 (PMC11530092; doi:10.1371/journal.pone.0313298)
Supplement: S1 Appendix — (DOCX) [file pone.0313298.s001.docx]

**Appendix 1: Search Strategy**

MEDLINE (Ovid)

Search conducted on August 4^th^ 2023

| **#** | **Query** | **Records retrieved** |
| --- | --- | --- |
| 1 | pharmaceutical preparations/ or controlled substances/ or exp dosage forms/ or exp drug combinations/ or drugs, essential/ or drugs, generic/ or exp nonprescription drugs/ or pharmaceutical preparations, dental/ or prescription drugs/ or prodrugs/ or exp solutions/ or synthetic drugs/ or exp "pharmacological actions (non mesh)"/ or drug industry/ or drug therapy/ | 5,578,371 |
| 2 | sd.fs. | 71,185 |
| 3 | 1 and 2 | 10,713 |
| 4 | (shortage* or discontinuation* or supply disruption* or recall* or stock out*).tw,kf. | 200,360 |
| 5 | 3 and 4 | 1,093 |
| 6 | drug recalls/ or safety-based drug withdrawals/ | 565 |
| 7 | ((drug or drugs or medicine or medication* or vaccin*) adj5 (shortage* or recall* or stock out* or supply disruption* or market withdrawal* or market discontinuation*)).tw,kf. | 4,098 |
| 8 | ((safety or regulat*) adj3 (drug withdrawal or medication withdrawal)).tw,kf. | 24 |
| 9 | (discont* adj3 (drug or drugs or medication* or medicine) adj10 (safety or regulat*)).tw,kf. | 171 |
| 10 | 5 or 6 or 7 or 8 or 9 | 5,308 |
| 11 | drug utilization/ or exp prescriptions/ or drug administration schedule/ or exp drug prescriptions/ | 163,731 |
| 12 | ((drug or drugs or medication* or medicine) adj3 utilizat*).tw,kf. | 6,093 |
| 13 | utilization trend*.tw,kf. | 547 |
| 14 | (prescribing adj3 pattern*).tw,kf. | 4,777 |
| 15 | practice patterns, pharmacists'/ or practice patterns, dentists'/ or practice patterns, physicians'/ | 69,533 |
| 16 | ((medication* or medicine or drug or drugs) adj3 dispensing pattern*).tw,kf. | 32 |
| 17 | prescription*.tw,kf. | 127,651 |
| 18 | (utilization pattern* or "pattern* of utilization" or treatment pattern* or "drug use").tw,kf. | 64,678 |
| 19 | or/11-18 | 379,999 |
| 20 | 10 and 19 | 634 |
