# Supplementary material for: Impact of supply chain disruptions and drug shortages on drug utilization: A scoping review protocol
Source: PLoS One. 2024 Nov 1;19(11):e0313298. doi: 10.1371/journal.pone.0313298 (PMC11530092; doi:10.1371/journal.pone.0313298)
Supplement: S2 Appendix — (DOCX) [file pone.0313298.s002.docx]

**Appendix 2: Targeted Website Search**

**Websites identified through targeted website search**

| **#** | **Website name/organization** | **Link** |
| --- | --- | --- |
| 1 | Health Canada | https://search.open.canada.ca/opendata/?_ organization _limit=0&organization=hc-sc |
| 2 | Canadian Agency for Drugs and Technologies in Health (CADTH) | https://www.cadth.ca/search |
| 3 | Health Quality Ontario (HQO) | https://www.hqontario.ca/Evidence-to-Improve-Care/Health-Technology-Assessment |
| 4 | Canadian Institute for Health Information (CIHI) | https://www.cihi.ca/en/access-data-and-reports |
| 5 | Government of Canada Publications | https://publications.gc.ca/site/eng/home.html |
| 6 | Canadian Pharmacists Association (CPA) | https://www.pharmacists.ca/ |
| 7 | US Food and Drug Administration (FDA) | https://www.fda.gov/ |
| 8 | American Society of Health System Pharmacists (ASHP) | https://www.ashp.org/ |
| 9 | Office of the Assistant Secretary for Planning and Evaluation (APE) | https://aspe.hhs.gov/ |
| 10 | Ministry of Health Ontario | https://www.ontario.ca/page/ministry-health |
| 11 | Ontario Health | https://www.ontariohealth.ca/ |
| 12 | Public Health Ontario | https://www.publichealthontario.ca/ |
| 13 | European Medicines Agency (EMA) | https://www.ema.europa.eu/en |
| 14 | Therapeutic Goods Administration (TGA) | https://www.tga.gov.au/ |
| 15 | Organisation for Economic Co-operation and Development (OECD) | https://www.oecd.org/health/ |
| 16 | International Pharmaceutical Federation (FIP) | https://www.fip.org/ |
