# Supplementary material for: Impact of supply chain disruptions and drug shortages on drug utilization: A scoping review protocol
Source: PLoS One. 2024 Nov 1;19(11):e0313298. doi: 10.1371/journal.pone.0313298 (PMC11530092; doi:10.1371/journal.pone.0313298)
Supplement: S3 Appendix — (DOCX) [file pone.0313298.s003.docx]

**Appendix 3: Data Extraction Form**

| **General Article Information** | |
| --- | --- |
| Title |  |
| First Authors |  |
| Journal |  |
| Year |  |
| DOI |  |
| Country(s) |  |
| Continent |  |
| Funding source(s) |  |
|  | |
| **Study Overview** | |
| Study design |  |
| Length of Follow-Up |  |
| Primary study aim/objective |  |
|  | |
| **Data Sources** |  |
| Data sources used |  |
| Healthcare setting |  |
| Type of drug use data used (i.e., dispensing, purchasing, prescribing, etc.) |  |
|  |  |
| **Methodology** |  |
| Data analysis |  |
| Statistical models |  |
| Covariates measured |  |
|  |  |
| **Results** | |
| **Drug characteristics** | |
| Drug(s) studied |  |
| Drug class |  |
| Formulation(s) if applicable |  |
| Dosage |  |
| Indication |  |
| Market Structure |  |
|  |  |
| **Shortage Characteristics** | |
| Shortage status |  |
| Start/End dates |  |
| Reason |  |
| Duration |  |
|  | |
| **Outcomes** | |
| Changes in drug use |  |
| Major findings |  |
| Conclusions |  |
|  | |
