# Supplementary material for: Impact of supply chain disruptions and drug shortages on drug utilization: A scoping review protocol
Source: PLoS One. 2024 Nov 1;19(11):e0313298. doi: 10.1371/journal.pone.0313298 (PMC11530092; doi:10.1371/journal.pone.0313298)
Supplement: Attachment — Submitted filename: Response to Reviewers.docx [file pone.0313298.s005.docx]

July 10^th^, 2024

Dr. Udoka Okpalauwaekwe

Academic Editor

PLOS One

Re: PLOS ONE - Decision on Manuscript PONE-D-23-29159

Dear Dr. Okpalauwaekwe,

We are delighted to learn of your journal’s interest in publishing our work and are grateful for the further suggestions provided. We have made the suggested changes to the manuscript based on the thoughtful suggestions from the reviewer’s comments. A point-by-point response to the comments is provided below.

Sincerely,

Araniy Santhireswaran

**Reviewer Comments to the Author**

**Reviewer #1**

1. **The authors should describe in more detail the logic of only including observational studies and excluding RCTS, etc. I would anticipate most of the studies will be observational in nature but it would be beneficial to the reader to understand the logic of excluding other types of studies as they have mentioned.**

*Thank you for this suggestion, we agree that explaining our reasoning will be helpful for readers, so we have added language about that in the eligibility criteria section and more clearly communicated our reasoning.*

*Change: For this scoping review, published and unpublished observational studies such as cohort, case-control and self-controlled designs, will be included. Our review question focuses on the impacts of drug shortages on drug use in large populations. Therefore, case series and case reports will be excluded since they only recount a few occurrences of the outcome instead of analyzing large populations. Moreover, randomized control trials (RCTs) will be excluded given that randomization is infeasible for our review question, and we aim to better understand the real-world impact of shortages. Literature reviews (i.e. systematic, scoping, narrative, etc.), meta-analyses, study protocols, opinion pieces (i.e. editorials, commentaries, letters, etc.), conference abstracts, and studies with unavailable full-text articles will be excluded due to the lack of primary research analysis.*

2. **The authors have not clearly stated how the data will be made available in the future**

*Thank you for pointing this out. We added a statement about data availability and highlighted this in our data disclosure.*

*Change: Relevant data from the extraction will be presented in appropriate tables and figures to showcase existing evidence and emphasize knowledge gaps in literature. All data will be made available in the supplemental tables of the final published manuscript or in an OSF project space with the link shared in the manuscript.*

3. **Citations: There are certain times when the citations are placed before the period of a sentence and other times where it is placed after. Please revise this to keep it uniform and in line with PLOS formatting.**

*Thank you for bringing this to our attention, we have ensured the superscript formatting is consistent throughout the manuscript.*

4. **My preference would be that the protocol ends with the brief discussion and impact session to once again revision the potential impact of this work (i.e how it will change practice and/or policy). I believe this would strengthen the protocol. The authors already provide some of this information in the introduction section but having it at the end of the paper may be of benefit too.**

*This is a great suggestion, thank you. We have now incorporated a significance section at the end of the manuscript.*

*Added:*

*Significance*

*Drug shortages continue to impair care delivery, patient clinical outcomes and the healthcare system. Understanding the extent, frequency and severity of drug shortages can help inform important policy decisions aimed at mitigating shortages. Currently there is limited research on the impact of drug shortages on patient drug use and access, and this proposed scoping review will summarize commonly studied shortages and their associated characteristics, settings and regions these studies have been conducted and most importantly the extent of the impact on use and access. It is crucial to compare the impacts of different shortages since not all shortages manifest similarly and have equal impacts. The findings from this proposed scoping review will inform future drug shortage research aimed at guiding drug shortage frameworks and policy-making decisions.*

**Reviewer #2**

1. **While literature reviews are listed in the exclusion criteria, will literature reviews be checked to see if they include any eligible observational studies that may have been missed by the search strategy?**

*Thank you for this suggestion. We will be checking the references list for some selected literature reviews in the field as a part of the reference hand search. We have updated this in the methods as well.*

*Change: A structured search will be conducted in MEDLINE and EMBASE. A grey literature search will be conducted using grey literature databases, a targeted website search, and the Google search engine. A supplementary hand-search of reference lists of included articles and any relevant reviews identified in screening will be conducted to identify any additional relevant articles that may have been missed by the search strategy.*

2. **Only searching MEDLINE and EMBASE may not be adequate for a comprehensive scoping review. The search strategy would be improved by searching at least one broad interdisciplinary database such as Scopus and at least one pharmaceutical literature database such as the International Pharmaceutical Abstracts database.**

*Thank you for this comment. We acknowledge that three databases are the standard for a scoping review. However, given that our question is very specific and will rely on a data collection strategy that goes beyond published literature, we decided that adding a broad interdisciplinary database, like Scopus or Web of Science, would make our published literature searches too sensitive for this question and we wanted to focus more of our data collection efforts on the grey literature searches. We felt that the two largest biomedical databases MEDLINE and EMBASE, which has the pharmaceutical focus, combined with TRIPPro which was included as part of our grey literature search, but does search for published literature combined with the supplemental reference tracking searches will be sufficient for gathering the published literature for this scoping review. We consulted our librarian who is also a co-author and tested searches in Web of Science and Pharmaceutical Abstracts to arrive at this conclusion. We felt that this search strategy, that has more of an emphasis on grey literature, compared with the guidance for a standard systematic review search, allows us to more fulsomely search for and answer our question. However, we realize that the lack of a third interdisciplinary database is a limitation and we have made changes to make this clear.*

*Change: A structured search will be conducted in MEDLINE and EMBASE. A grey literature search will be conducted using grey literature databases, a targeted website search, and the Google search engine. We understand that searching 3 databases is the standard for a scoping review and including a broad interdisciplinary database will improve our search. However, given that our search strategy is very specific we decided to conduct an extensive grey literature search instead of including a third broad interdisciplinary database. We acknowledge that this is a potential limitation of our scoping review.*

3. **The grey literature search strategy requires further development and explanation. What is the rationale for including each grey literature source? Why are preprints and conference proceedings not being searched as part of the grey literature strategy?**

*Thank you for these suggestions. For the purposes of this grey literature search, we want to focus on finding the research disseminated through alternative means of publication, for example, the reports that many government and healthcare organizations may produce but will not publish in a traditional article and the search strategy was designed with this in mind. We have elaborated on the rationale for the grey literature search by explaining why we chose these sources. As for your suggestion about preprints, the papers we aimed at including are not deposited in pre-print servers so we felt that searching these would not provide many relevant studies. Also, conference abstracts to be included as a standalone are not substantial enough as they lack the details of a full paper and can often present preliminary data that could change by the time the research is completed. Therefore, our grey literature databases focus on studies conducted by government and healthcare organizations primarily, and we have made changed to further explain this in the manuscript.*

*Change: The targeted website search will be conducted using Advanced Search on Google to filter for relevant uploaded reports and files. A list of websites is provided (see Appendix 2). Many government and healthcare organizations lead drug shortage related research aimed at guiding policy decisions; therefore, government and healthcare websites were primarily included in the targeted website search.*
