# Supplementary material for: Impact of supply chain disruptions and drug shortages on drug utilization: A scoping review protocol
Source: PLoS One. 2024 Nov 1;19(11):e0313298. doi: 10.1371/journal.pone.0313298 (PMC11530092; doi:10.1371/journal.pone.0313298)
Supplement: Attachment — Submitted filename: Response to Reviewers.docx [file pone.0313298.s006.docx]

September 16^th^, 2024

Dr. Udoka Okpalauwaekwe

Academic Editor

PLOS One

Re: PLOS ONE - Decision on Manuscript PONE-D-23-29159

Dear Dr. Okpalauwaekwe,

We grateful for the further suggestions provided. We have made the suggested changes to the manuscript based on the suggestions from the reviewer’s comments. A point-by-point response to the comments is provided below. In the manuscript with tracked changes we have highlighted all changes made since the original submission.

Sincerely,

Araniy Santhireswaran

Reviewers' comments:

Reviewer's Responses to Questions

**Reviewer #2:**

1. **The revision explains the rationale for only searching two databases (MEDLINE and EMBASE) and mentions that the search strategy was tested on other databases in order to decide not to include them. The data from the testing of the search strategy should thus be included as a supplement with the manuscript.**

*Thank you for this suggestion. We acknowledge that only searching two databases is not the standard for a scoping review, and we did explore other databases including Web of Science and Scopus before reaching this decision. However, this decision process was not documented as it was a part of our preliminary brainstorming and was not done in a systematic manner. We were not aware that we should document this process since the PRISMA guidelines do not ask for this level of reporting. We understand how the wording in the manuscript can make it seem like it was a systematic process, so we have modified the wording for it to be clearer.*

*Change: We understand that searching 3 databases is the standard for a scoping review and including a broad interdisciplinary database will improve our search. However, given that our search strategy is very specific we decided to conduct an extensive grey literature search instead of including a third broad interdisciplinary database. We conducted preliminary explorations of our search in MEDLINE, EMBASE, Web of Science, Scopus and Pharmaceutical abstracts and decided that both MEDLINE and EMBASE were sufficient for our review.*

2. **The additional detail on the grey literature search strategy now included in the manuscript does make the methods more reproducible. The search methods section includes an incomplete sentence: "As interdisciplinary databases Web of Science and Pharmaceutical Abstracts did not bring in many relevant articles given our niche topic." Revising this sentence in the search methods section, and including the testing data for the search strategy, will make the protocol acceptable for publication.**

*Thank you again for the feedback on adding more detail on the grey literature search, we believe this has increased reproducibility of the review. Also, thank you for pointing out the incomplete sentence, we have modified it now.*

*Change: Given our niche topic, interdisciplinary databases such as Web of Science, Scopus and Pharmaceutical Abstracts did not bring in many relevant articles. We acknowledge that including only 2 databases is a potential limitation of our scoping review.*
