# Supplementary material for: Impact of supply chain disruptions and drug shortages on drug utilization: A scoping review protocol
Source: PLoS One. 2024 Nov 1;19(11):e0313298. doi: 10.1371/journal.pone.0313298 (PMC11530092; doi:10.1371/journal.pone.0313298)
Supplement: Attachment — Submitted filename: Response to Reviewers.docx [file pone.0313298.s007.docx]

October 12th, 2024

Dr. Udoka Okpalauwaekwe

Academic Editor

PLOS One

Re: PLOS ONE - Decision on Manuscript PONE-D-23-29159

Dear Dr. Okpalauwaekwe,

We grateful for the further suggestions provided. We have made the suggested changes to the manuscript based on the suggestions from the reviewer’s comments. A point-by-point response to the comments is provided below. In the manuscript with tracked changes we have highlighted all changes made since the original submission.

Sincerely,

Araniy Santhireswaran

Reviewers' comments:

Reviewer's Responses to Questions

**Reviewer #2:**

Two minor revisions/clarifications needed before the protocol is ready for publication:

1. **Search methods: The search methods section gives a rationale for including two databases (MEDLINE and EMBASE) and a grey literature search instead of a broad interdisciplinary database. However, stating that "a broad interdisciplinary database will improve our search" is confusing. If it would improve the search to include a broad interdisciplinary database, why not do so? Instead, it could be clearer to just give the rationale for why the two disciplinary databases and targeted grey literature strategy were selected, rather than stating that the search would be improved by including an interdisciplinary database and then not doing so.**

*Thank you for this suggestion. We agree that this statement can be confusing. We have made appropriate changes.*

*Changes: We understand that searching 3 databases is the standard for a scoping review. However, given that our search strategy is very specific we decided to conduct an extensive grey literature search instead of including a third broad interdisciplinary database.*

2. **Data availability: The data availability statement states that all data from the review will be made available. However, it does not specify how or where the data will be made available.**

*Thank you for pointing this out. We have specified how the data will be made available in the data availability statement.*

*Changes: All data will be made available in the supplemental tables of the final published manuscript.*
